# Case of Disease of the Left Kidney, Which Terminated Fatally

**Published:** 1828-10

**Authors:** J. Bryant

**Affiliations:** Surgeon Accoucheur.


### DISEASE OF THE KIDNEY.

### *Case of Disease of the left Kidney, which terminated fatally.*

By J. Bryant, Esq. Surgeon Accoucheur.

Mrs. T—, aged twenty-five years, a healthy looking woman, fair complexion, was, four days after a tedious labour, (in which both the fœtuses and secundines were materially assisted in their expulsion by secale cornutum,) seized with severe pain in the left side, about the sigmoid flexure of the colon. Up to this period she was doing extremely well; lochial discharges natural, and plentiful secretion of milk. The pain was increased by pressure. Recollects, about two years since, being affected in a similar manner; which she attributed to have been in consequence of carrying a heavy weight up stairs.

Pulse frequent, full, and hard, with general pyrexial symptoms; the bowels had been evacuated the previous day by salts and senna.

Ordered to be bled to ℥xiv. Twenty leeches to part affected; and two pills of Calomel, Antimony, and Opium directly, and a dose of Castor-oil in four hours.

Six o'clock p.m.—Bowels have been freely relieved; pain and fulness much diminished; has slept three hours, and could turn on either side; pulse eighty-four, and soft; skin moist.

Take, every five hours, Sulphate of Magnesia, Tartarised Antimony, and a pill of Extract of Henbane with each dose of the mixture. Dry fomentations of heated malt to the side; and farinaceous diet.

These remedies were continued for the next five days, when she became convalescent, and continued tolerably well up to the 15th April, when my attendance was again requested. She now complained of uneasiness of the same side, which she ascribes to over-exertion. Has applied a blister, which is now acting. Pulse nearly natural, with little or no febrile excitement; bowels constipated.

Ordered recumbent position and aperient medicine.

17th.—Bowels relieved; less uneasiness, but has vomited during the night, stomach continuing irritable.

Saline medicines in a state of effervescence, with small doses of Tincture of Opium, were given.

18th.—The stomach is now quiet. Blister nearly healed; but there is an evident fulness and hardness over the seat of pain, which is increased by pressure. Pulse frequent, and somewhat fuller.

Ordered fourteen leeches to the part; and a draught with Rhubarb and Tartrate of Potass directly; and a mixture of Tincture of Henbane and Infusion of Linseed every five hours.

21st.—Pain relieved; pulse ninety, and less full; bowels open

Repeat the leeches and saline medicines, with Extract of Henbane, every four hours.

23d.—Fulness and hardness increasing, and extending obliquely towards the right hypochondrium, and downwards to the pubes.

Repeat the leeches, and continue remedies.

24th.—Pain relieved. The tumor reaches from the pubes, and extends to and under the margin of the ribs on the left side; pain more dull and obtuse. Had a smart rigor this morning. Urinates freely and without pain, but the secretion emits an odour similar to animal broth in a state of decomposition, yet not turbid. She was now seen by several medical friends, some of whom were of opinion it was a suppurative spleen; others, an abscess in the left ovarium. Dr. Nevinson, who saw her at this time, conjectured it might be the kidney. Cataplasms with conium were now applied to the tumor. Pulse quick, small, and hard; bowels regular; has not rested.

Opiate at bedtime.

26th.—Blister over the tumor, and five grains of the Soap-pill, with opium and saline mixture, every five hours.

28th.—Blister has afforded some relief. Has had a good deal of bilious vomiting for the last three or four days.

Carbonate of Potass and Magnesia, with lemon-juice, every five hours; with an opiate pill at bedtime.

80th.—There is a perceptible deep-seated fluctuation about the centre of the tumor. Cataplasms with conium have been continued, from which she experienced considerable relief.

May 3d.—Still urinates freely, but it emits the same putrescent animal odour before remarked.

Take, every four hours, the decoction of Uva Ursi, with the Subcarbonate of Soda.

5th.—A considerable quantity of pus has been passed with the urine this morning. Tumor in the side considerably diminished.

Sulphate of Quinia, with Aromatic Confection, was now given every five hours.

7th.—Large quantities of pus continue to be discharged with the water, but without pain. Tumor is no longer perceptible, the abdomen being quite flaccid; no pain on pressure. Diarrhœa supervened this morning. Pulse quick and feeble; body much attenuated; animal spirits good.

Cusparia Bark was substituted for the Cinchona; and a large plaster of Albanum was applied over the left side, and a broad belt placed comfortably tight around the abdomen.—Diet, rice, chicken broth, and beef tea.

9th.—Diarrhœa still continuing, ordered Chalk mixture, with compound Powder of Kino, after every loose motion.

14th.—Diarrhœa having continued, with intermissions, since the 9th instant, half a grain of Superacetate of Lead, and half a grain of Opium, were given every four hours. The relief afforded by this medicine, both as regards the alvine and urinary excretions, was strikingly marked; but colicky pains supervening on the 24th, it was thought prudent to discontinue the lead.

29th.—Diarrhœa having again become troublesome, the white decoction, with compound Powder of Kino, was administered.

31st.—Diarrhœa somewhat alleviated.

Continue remedies, with Extract of Cascarilla and Black Pepper in pills.

June 8th.—Diarrhœa still continues, the dejections often appearing puriform.

Small doses of Sulphate of Copper with Opium were now exhibited.

15th.—The diarrhæa has been considerably diminished since the administration of the Sulphate of Copper, but was discontinued this morning, in consequence of a return of the bilious vomiting.

29th.—Very little medicine was given up to this period. The tongue and fauces have assumed an apthous appearance. Diarrhœa occasionally troublesome; urine sometimes clear, at others turbid; the putrescent odour, which formerly emitted, has never been perceptible since the discharge of matter per urethra. Subborate of Soda, with Tincture of Opium and Infusion of Linseed, were given every four hours. These remedies were continued, with trifling alterations, up to the day of her dissolution, which took place on the 21st July.

During the last four days of her existence, she was seized with symptoms of cerebral derangement; pulse, which was before quick, small, and weak, now became comparatively full and hard; she could with difficulty be kept in bed; contracted pupil, and other symptoms of strong cerebral excitement.

*Post-mortem examination,* eighteen hours after death.—On proceeding to examine the body, the left side presented a dark surface, similar in appearance to what takes place in cases of internal mortification; and, on laying open the abdomen, the whole internal viscera were found considerably shrunk and displaced; the stomach being pushed upwards, having the lower opening in a direct line from above with the cardiac portion; liver preternaturally large; colon higher than usual, and the duodenum drawn to the left side. In the gall-bladder were found three biliary calculi, and one in the cystic duct. The left kidney was found much enlarged and discoloured, the pelvic portion of which was partially filled with dark, ill-conditioned pus, together with fourteen calculi, the size of hazel nuts, and had evident marks of having been the sac of an immense abscess, the contents of which had been discharged partly by the ureter, and partly by an opening in the duodenum, between which portion of intestine and the sac a communication was formed by ulceration. A layer of coagulable lymph covered the bladder externally, but in other respects was perfectly healthy.

*Edgeware Road; August* 1828.

